# Physicochemical Stability of Doravirine (Pifeltro^®^): Characterization of Main Degradation Products and Assessment of Stability of Tablets Repackaged in Single-Dose Unit Containers

**DOI:** 10.3390/pharmaceutics16080977

**Published:** 2024-07-24

**Authors:** Moïse Houssen, Philippe-Henri Secretan, Loup Nobilet, Kilian Jossot, Laura Guichard, Cédric Mwamba, David Ngy, Lamia Hassani, Audrey Solgadi, Marie Antignac, Bernard Do, Helga Junot, Hassane Sadou Yayé

**Affiliations:** 1 Department of Pharmacy, Hôpitaux Universitaires Pitié-Salpêtrière, AP-HP, 75013 Paris, France; azadmoise@gmail.com (M.H.); loup.nobilet@aphp.fr (L.N.); laura.guichard@aphp.fr (L.G.); cedric.mwamba@aphp.fr (C.M.); davidngy97@gmail.com (D.N.); lamia.hassani@aphp.fr (L.H.); marie.antignac@aphp.fr (M.A.); helga.junot@aphp.fr (H.J.); 2 Matériaux et Santé, Université Paris-Saclay, 91400 Orsay, France; 3 UMS-IPSIT SAMM Facility, Université Paris-Saclay, Inserm, CNRS, Ingénierie et Plateformes au Service de l’Innovation Thérapeutique, 91400 Orsay, France; audrey.solgadi@universite-paris-saclay.fr; 4 Université Paris Cité, Inserm, PARCC, 75015 Paris, France; 5 Clinical Pharmacy Department, Gustave Roussy Cancer Campus, 94805 Villejuif, France; bernard.do@universite-paris-saclay.fr; 6 Institut des Sciences Moléculaires d’Orsay, Université Paris-Saclay, CNRS, 91405 Orsay, France

**Keywords:** doravirine, mass spectrometry, degradation products, structural elucidation, stability, repackaging

## Abstract

Doravarine (DOR) is an antiviral drug with a marketed authorization for the management of occupational blood and body fluid exposure. The currently existing packaging, consisting of multiple unit bottles comprising 30 tablets, is not fully appropriate for daily nominative dispensing at the hospital. This study aims at assessing the impact of the change in packaging on the key attributes of the drug: assay, impurity profile, and dissolution. As the first step, which is not fully depicted in the literature, the main potential impurities that could appear during storage (i.e., degradation products (DPs) of DOR) were characterized using a forced degradation protocol followed by an LC-MS/MS analysis. These results paved the way for in silico toxicological assessment and targeted degradation product profiling. Based on this study, the assessment of the implication of repackaging on the formation of DOR’s degradation products should be a primary focus.

## 1. Introduction

The current management of occupational blood and body fluid exposure within our hospital entails rilpivirine-based treatment within 48 to 72 h, pending consultation with an infectious disease specialist. Recently, a new drug, doravirine (DOR), 3-Chloro-5-({1-[(4-methyl-5-oxo-4,5-dihydro-1H-1,2,4-triazol-3-yl)methyl]-2-oxo-4-(trifluoromethyl)-1,2-dihydro-3-pyridinyl}oxy)benzonitrile), a non-nucleoside reverse transcriptase inhibitor (NNRTI), has been authorized for this indication. This new drug has the advantage of being better tolerated than rilpivirine, displaying fewer interactions with other medications, and having less significant food effects on bioavailability [1,2].

The commercial packaging of DOR, currently consisting of bottles containing 30 tablets [3], is less adapted than single-dose unit containers for the appropriate management of occupational blood and body fluid exposure (BBFE) and for daily nominative dispensing at the hospital [4]. Thus, to respond to that need, hospital pharmacists may resort to repackaging the tablets in single-dose units. However, there are no available data regarding the stability of the tablets once repackaged in single-dose unit dosage containers, although it has been shown that repackaged drugs may fail one or more chemical and/or physical testing [5].

To anticipate the potential issues regarding stability, ICH Q1A [6] recommends assessing the stability of the drug under stress conditions. From there, the knowledge of the structure of the degradation products can help to highlight the main mechanisms through which the drug may be degraded. Gaining access to the degradation products enables the design of a stability-indicating method, which can then be applied to follow up the stability of the drug under formal storage conditions [6]. Unfortunately, no data regarding the intrinsic stability of DOR could be found in the literature and in the European Assessment Report (EPAR) of Piferto^®^ [7]. The recommendation regarding Pilfetro^®^ tablets stated in the EPAR “Store in the original bottle and keep the bottle tightly closed in order to protect from moisture. Do not remove the desiccant.” [7] underlines that DOR may be susceptible to degradation. The same information regarding DOR stability is found in the EPAR of Delstrigo^®^ [8], which consists of tablets comprising DOR, lamivudine, and tenofovir disoproxil fumarate.

Thus, this study aimed to evaluate the physicochemical stability of DOR under stress conditions, with the view to highlight the main degradation products and assess if tablets repackaged in single-dose unit containers and stored for 6 months at 25 °C still complied to requirements in terms of assay, degradation product content, and dissolution test. To that end, a forced degradation study of DOR was implemented with the view of highlighting its main degradation products by liquid chromatography coupled with diode array detection. Their structural elucidation was then carried out by in-depth interpretation of high-resolution mass spectra, paving the way for the mutagenicity assessment of these degradation products. Tablets of DOR stored for 6 months under long-term conditions (at 25 °C and 60% relative humidity) were then assessed in terms of dissolution, disintegration, assay, and impurity profiling.

## 2. Materials and Methods

### 2.1. Reagents and Raw Materials

A 100 mg quantity of film-coated tablets containing DOR (Pifeltro^®^) were purchased from MSD (Puteaux, France), and doravirine standard reference was procured from Sigma Aldrich (St. Quentin Fallavier, France). HPLC gradient reagents (acetonitrile and formic acid) were provided by VWR (Fontenay-sous-Bois, France). Ultrapure water was produced by the Q-Pod Milli-Q system (Millipore, Molsheim, France). Hydrogen peroxide (H_2_O_2_) 30% *v*/*v* was supplied by Carlo Erba SDS (Val de Reuil, France). Individual PVC reconditioning blisters, responding to class A requirements of USP monograph <671> CONTAINERS—PERFORMANCE TESTING, and proper labels were procured from Pero’S (Lyon, France).

### 2.2. Instrumentations and Analytical Procedures

DOR content variation and its potential degradation product apparition over time were monitored using a reversed-phase liquid chromatography (LC) system (Dionex, Les Ulis, France), consisting of a quaternary pump, a vacuum degasser, a photo-diode-array (PDA) detector, and an autosampler piloted by Chromeleon^®^ software version 6.80SR11 (Dionex, Les Ulis, France). A stability indicating method suitable for drug quantification as well as for degradation product determination was developed and optimized according to ICH Q2 (R1) [9]. To this end, DOR solutions were exposed to stress under thermal conditions (80 °C for 2 days), hydrolysis (acid 1 N and basic 0.1 N conditions for 2 days), oxidative condition (3.5% H_2_O_2_ for 2 days), and photolysis (for 2 h) using a Q-SUN XE-1 Xenon test chamber (LX-5080, Q-LAB, Westlake, CA, USA) following the recommendation of ICH Q1B [10] (Supplementary Materials (Table S1)). The light intensity delivered was 1.50 W.m^−2^ at 420 nm (UV irradiance from 300 to 400 nm: 66.5 W.m^−2^; illuminance: 119.6 klx). The temperature was set at 20 °C. Samples were prepared in triplicate for each condition.

Separation was performed with Zorbax SB-C8 250 mm × 4.6 mm; a 5 μm column set at 20 °C; and a mobile phase flow rate of 1 mL·min^−1^. A gradient mode combining 0.1% (*v*/*v*) formic acid added to both solvents, i.e., pure water (solvent A) and acetonitrile (solvent B), was used to separate and detect compounds over a wide range of polarities. The gradient program was set as follows: 0–2 min: 95% A; 2–30 min: 95 → 0% A; and 30–35 min: 95% A. The detection was set at 222 nm. The method was validated by evaluating linearity, specificity, selectivity, precision, accuracy, and LOD and LOQ parameters (Supplementary Materials (Table S2)).

The identification of the degradation products was performed by LC-HR-MS with the formerly described LC system coupled to an electrospray-LTQ-Orbitrap Velos Pro system consisting of a double linear trap followed by an orbital trap (Thermo Fisher Scientific, CA, USA). Analysis was carried out in the positive ion mode (ESI^+^), and the source voltage and the source and the capillary temperatures were set at 3.4 kV and 300 °C and 350 °C, respectively. The mass range of 105–1000 amu was used for preliminary high-resolution mass spectrometry (HR-MS) studies and that of 50–700 amu for HR-MSn studies. Instrumentation calibration was performed using the Pierce™ LTQ Velos ESI positive ion calibration solution (ThermoFisher, Waltham, MA, USA). The MS data were processed using Xcalibur^®^ software (version 2.2 SP 1.48, ThermoFisher, Waltham, MA, USA).

### 2.3. Experimental Protocol and Stability Study

The behavior of the drug product repackaged in individual cristal PVC reconditioning blisters; outside the bottle; and in the bottle simulating Pifeltro^®^’s Summary of manufacturers Product Characteristics (SmPC) recommendation were assessed. Pifeltro^®^ film-coated tablets were placed under the following storage conditions: (1) out of the bottle at room temperature (RT) (20 ± 2 °C); (2) in the bottle opened once daily at RT, consistent with the drug SmPC recommendation; (3) repackaged in PVC blister at RT; and (4) out of the bottle at 25 °C and 60% relative humidity (RH) for long-term studies (Supplementary Materials, Table S3).

In addition, solid-state stress studies were conducted on the powder from the tablets crushed at RT in a volumetric flask at 25 °C and on the tablets under accelerated storage conditions at 40 °C in a drying oven (Thermofisher, Waltham, MA, USA).

As DOR is practically insoluble in water [11], the tablets were first crushed, and then dissolved in acetonitrile. For the stability studies, for each condition, DOR content variation was assessed in triplicate on days 0, 1, 4, 7, 21, 30, 60, 90, and 180. Various parameters were monitored: color variation by visual examination, content variation by liquid chromatography (LC), based on a 5% threshold, and characterization of degradation products by liquid chromatography coupled with mass spectrometry (LC-HR-MS^n^). Tablet disintegration test was conducted on the samples stored in the blister at RT and at 40 °C, as per the European Pharmacopoeia recommendation of the Ph Eur Monogaph <2.9.1. Disintegration of Tablets and Capsules> using DIST-3 Triple Basket Tablet Disintegration Tester (Hainburg, Germany). The stroke height was set to 55 mm at 30 strokes/min.

### 2.4. Solubility Test

As active pharmaceutical ingredients could undergo polymorphic transformation during storage, and the difficulty of characterizing the polymorphic form within the drug product, led us to evaluate it indirectly by the mean of the solubility of the active ingredient within the drug product. The experiments were performed on a Cimarec-i Multipoint 6 Stirrer (Thermo Scientific, Les Ulis, France). Since DOR is practically insoluble in water [7], the evolution of its solubility in the tablets was evaluated in a water–acetonitrile mixture (50/50) at 20 °C. The control tablets are compared with those stored for 6 months in blister packs at room temperature (sample E1) and at 40 °C (sample E2). For each storage condition, a sample is taken at 5, 10, 20, 30, 45, 60, 90, 120, and 150 min, respectively, and then analyzed by the HPLC method described above.

### 2.5. Dissolution Testing

The dissolution tests were conducted with a Sotax AT7 Smart apparatus that complies with European Pharmacopoeia and USP guidelines. The paddle was set to 75 rpm, and 1000 mL of the dissolution medium consisting of 25 mM phosphate buffer (pH 6.8) with 3% *w*/*v* Polysorbate 80 (Sigma Aldrich; St. Quentin Fallavier, France) was used for testing the samples, in line with the conditions reported in the quality assessment of DOR’s tablets by the FDA’s Center for Drug Evaluation and Research (NDA 210806). The temperature was set at 37 °C. The test was conducted on six tablets, sampled after 45 min of dissolution, and then analyzed in duplicate using HPLC.

### 2.6. In Silico Mutagenicity Testing

In accordance with the ICH M7 guidelines [12], two computational methods were employed to evaluate the mutagenic potential of all identified degradation products. The first software used was Toxtree [13], a rule-based system. The second method was T.E.S.T. [14], which utilizes quantitative structure–activity relationships (QSARs).

## 3. Results

### 3.1. Forced Degradation Studies

#### 3.1.1. Stability Profile of DOR Based on Its Behavior under Different Stress Conditions

Under oxidative, acid, and thermal stress conditions, DOR exhibited stability, as no significant loss was observed, and no degradation products were detected.

**Figure 1:**
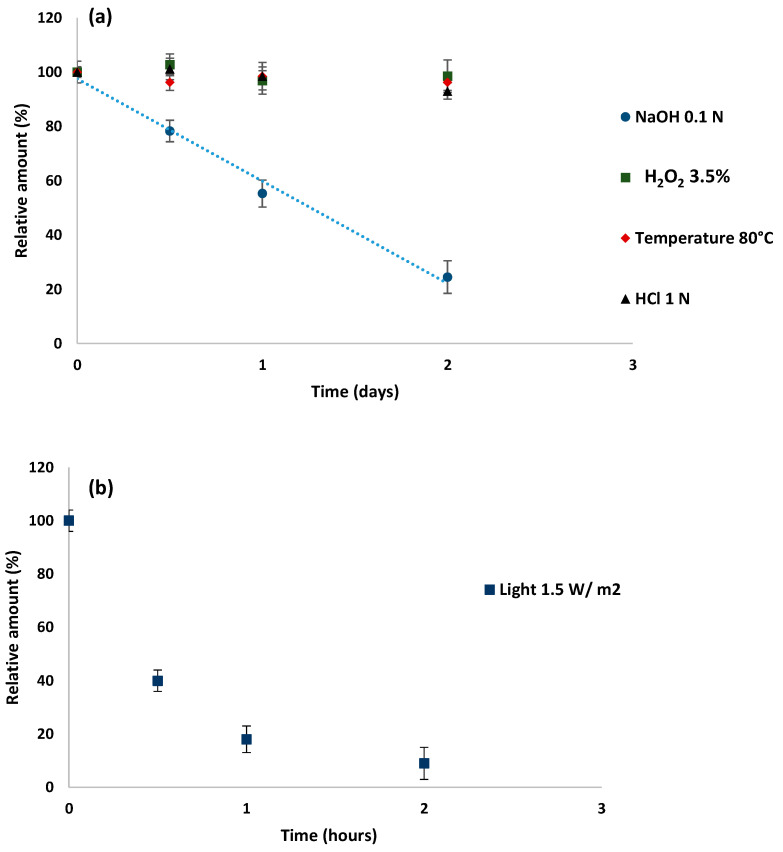
The effect of stress conditions on the kinetic degradation of DOR: (**a**) basic hydrolysis produced by 0.1 N NaOH (blue dots); thermal condition at 80 °C (red dots); oxidative condition produced by 3.5% H_2_O_2_ (green dots), and acidic hydrolysis produced by 1 N HCl (black dots); (**b**) photolytic condition.

In contrast, under photolytic or basic conditions, DOR underwent rapid degradation, as shown in Figure 1.

**Figure 2:**
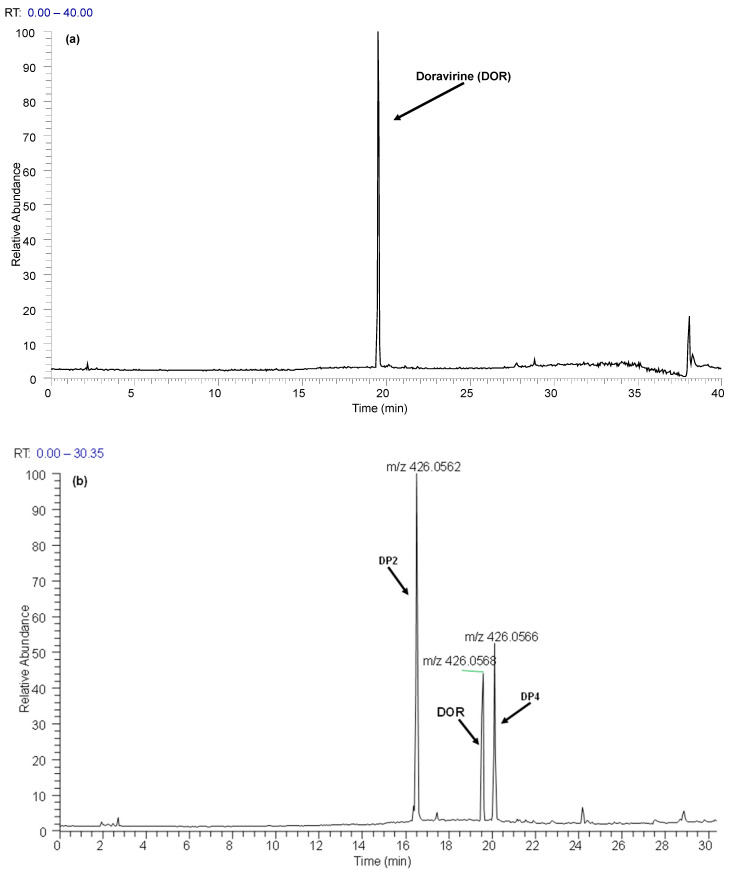

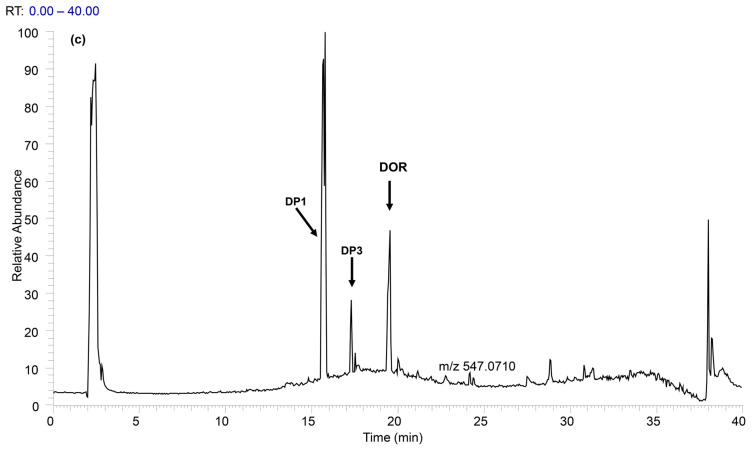
Total extracted ion chromatogram of a DOR solution (initial concentration = 25 μg/mL) unexposed to stress conditions: (**a**) DOR standard solution; (**b**) light weathering conditions; and (**c**) basic hydrolysis condition.

Analysis of solutions revealed two main different degradation products both under photolytic and under basic conditions. All degradation products detected using HPLC-UV were also confirmed using mass spectrometry detection (Figure 2). The structures of these degradation products were then determined by interpreting the HRMS^n^ mass spectra (see Section 3.1.2) at the corresponding retention times.

#### 3.1.2. Structural Elucidation of DOR Main Degradation Products 

All supportive mass spectra data are reported in the Supplementary Materials (Figures S1–S15). Accessing DOR’s main fragmentation routes is a key factor in helping the elucidation of its related compounds. As a result, the accurate mass data and the elemental composition of both the drug’s molecular ions and their related fragments were determined.

##### Fragmentation Pattern of DOR

**Figure 3:**
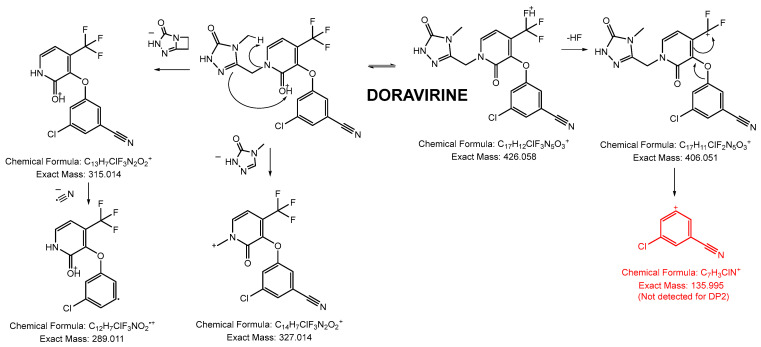
Fragmentation pattern of DOR.

Under LC-HRMS^2^ conditions, DOR (C_17_H_12_ClF_3_N_5_O_3_^+^) yielded five fragment ions whose accurate mass corresponded to characteristic loss. The fragmentation could be due to an ionization occurring at two different sites of DOR (Figure 3).

When the carbonyl of the central ring of the molecule is protonated (pKa = 0.34), a rearrangement occurs that results in the production of the base peak ion (315.014, C_13_H_7_ClF_3_N_2_O_2_^+^) by a neutral loss of the 1,3,4-triazabicyclo[3.2.0]hept-4-en-2-one moiety. Another neutral loss can occur by the cleavage of the bond between two of the rings of DOR, leading to the formation of C_14_H_7_ClF_3_N_2_O_2_^+^ (Figure 3).

Owing to the very low pKa of the functional groups of DOR, another site of protonation is the fluoride atoms of the molecule. This explains why C_17_H_11_ClF_2_N_5_O_3_^+^ is the second most intensely detected ion in the LC-HRMS^2^ spectra of DOR. The possibility of protonation of this site explains the formation of C_7_H_3_ClN^+^, which has not been detected for other DPs and was helpful for DOR’s structural elucidation.

##### Structural Elucidation of the Main Degradation Products Formed under Basic Conditions (DP1 and DP3)

**Figure 4:**
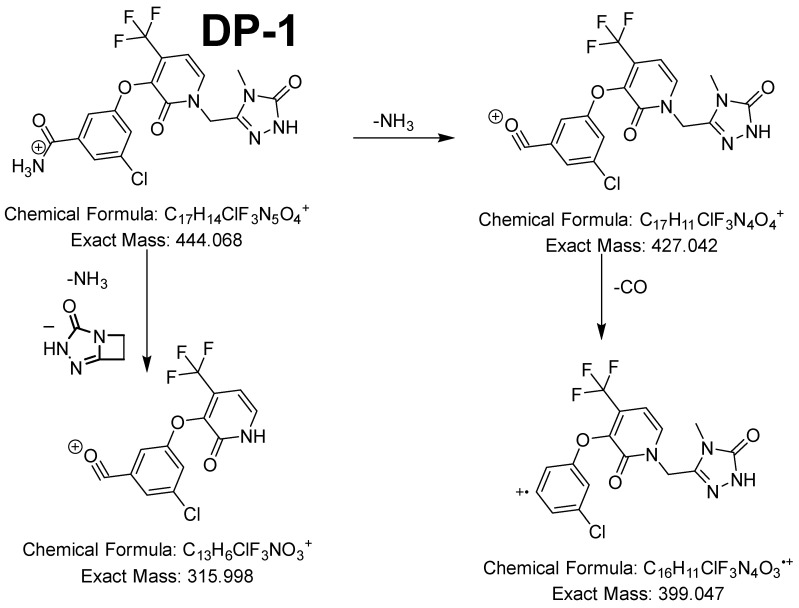
Fragmentation pattern of DP1.

The molecular ions DP1 and DP3 had accurate masses of 444.067 (Figure S5) and 445.052 (Figure S8), which correspond to C_17_H_14_ClF_3_N_5_O_4_^+^ (relative error = −3.70 ppm) and C_17_H_13_ClF_3_N_4_O_5_^+^ (relative error = −1.48 ppm). Based on their molecular formulas and the detection of ions with accurate mass identical to that of DOR, it could be inferred that their structures could be attributed to the hydrolysis that occurred at the cyano group. This was confirmed by the fact that the cyanide loss detected in the case of DOR was no longer detected for DP1 and DP3 but was replaced by the loss of ammonia and carbon monoxide (DP1, Figure 4) and by the loss of water and carbon monoxide (DP3, Figure S1).

##### Structural Elucidation of the Photodegradation Products (DP2 and DP4)

With an accurate mass of 426.057 (Figures S11 and S14), DP2 and DP4 displayed the same elemental composition as DOR (C_17_H_12_ClF_3_N_5_O_3_^+^). Based on the chromatographic profiles, where DP4 was detected after a significantly longer exposure to light, it can be assumed that DP4 was a secondary photodegradation product resulting from the degradation of DP2.

**Figure 5:**
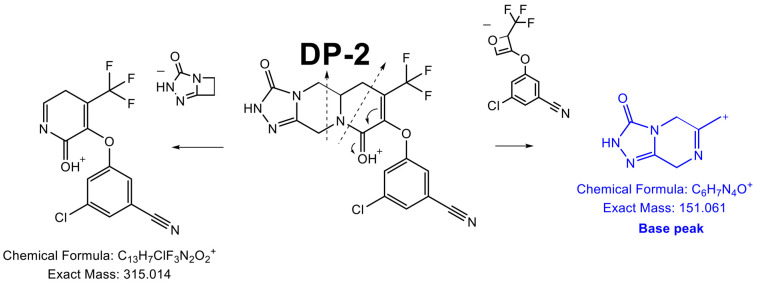
Fragmentation pattern of DP2.

DP2 base peak had an accurate mass (151.062, Figure S12) corresponding to C_6_H_7_N_4_O^+^ and not detected in the case of DOR and the other DPs. Based on this molecular formula and the structure of DOR, the presence of this ion resulted from the formation of an additional cycle in the molecule (Figure 5).

**Figure 6:**
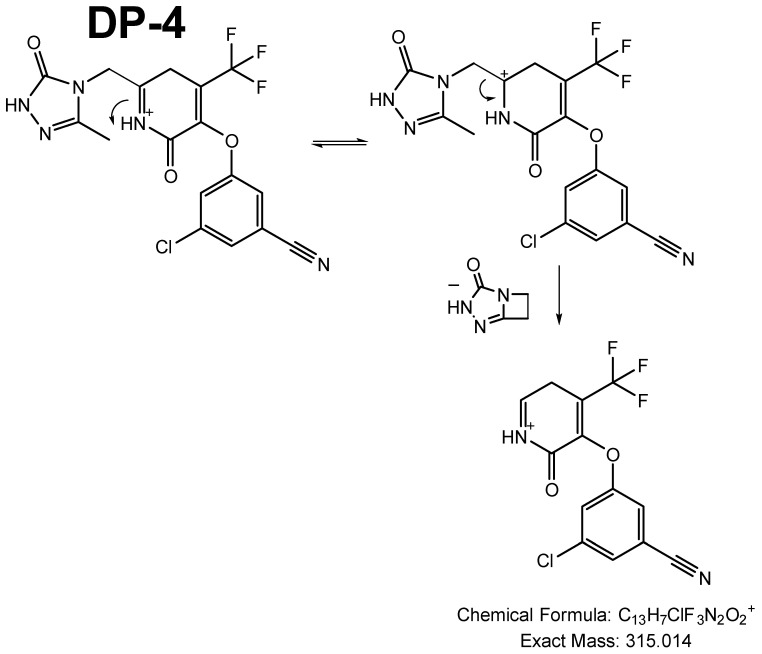
Fragmentation pattern of DP4.

Based on the fact that DP4 seemed to be formed by the degradation of DP2, it can be assumed that this photoproduct resulted from the reopening of the saturated ring of DP2, this ring being particularly susceptible to opening (Figure 6).

#### 3.1.3. In Silico Assessment of DOR’s Degradation Products

Structural elucidation of DOR’s degradation paved the way for the mutagenicity assessment. To that end, two approaches were used as per the ICH M7 recommendations. The results are reported in Table 1.

#### 3.1.4. Influence of Storage Parameters on DOR Solubility

**Figure 7:**
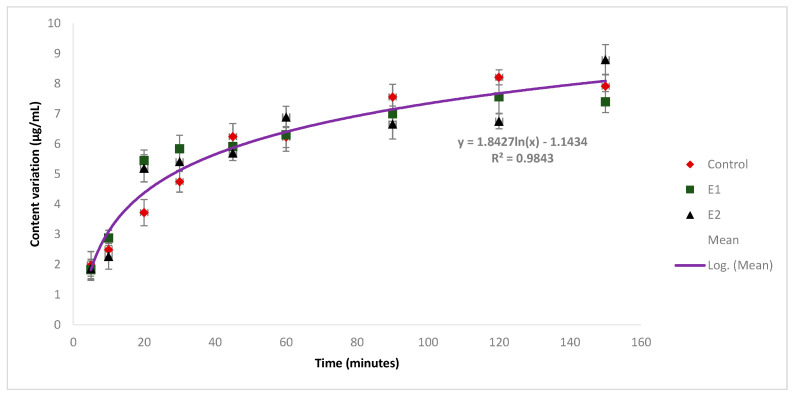
Solubility of DOR in a water–acetonitrile mixture (50/50) at 20 °C: control sample (red dots); sample E1 at RT (green dots); and sample E2 at 40 °C (black dots).

As polymorphism has been reported in DOR’s EPAR [7], it was important to assess if conditions such as temperature or humidity could affect DOR’s solid-state behavior in tablets. Based on previous studies, where authors investigated polymorphic changes in drugs in tablets by using solvent systems [15], tablets were stored under various conditions, and the solubility of DOR in the water–acetonitrile mixture (50/50) was compared (Figure 7). The result is a fitted regression line y = 1.8427ln(x) − 1.1434 (R^2^ = 0.9843). The data displayed similar solubility data between the control sample and those stored for 6 months at room temperature and 40 °C. As a result, the storage conditions did not seem to alter DOR’s solid-state properties.

### 3.2. Assessment of Stability of Repackaged Tablets

#### 3.2.1. Assay of DOR of Tablets

**Figure 8:**
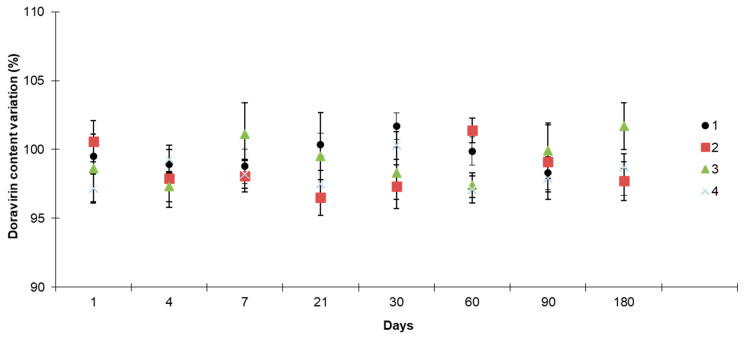
DOR content variation over time: (1) repackaged in PVC blister at RT (20 ± 2 °C); (2) in the bottle opened once daily at RT; (3) out of the bottle at room temperature (RT); and (4) at 25 °C and 60% relative humidity.

DOR content variation in the tablets is less than 5% regardless of the storage condition from D0 to D180 (Figure 8).

#### 3.2.2. Impurity Profiling of Tablets

Monitoring the impurity profiles of repackaged tablets stored at 25 °C for 6 months revealed no additional peaks, indicating that repackaging did not affect the impurity profile of DOR in the tablets.

The four degradation products (DP1–DP4) characterized in Section 3.1.2. were also searched by targeted mass spectrometry by performing extracted ion chromatograms on the solutions of tablets analyzed as per the conditions mentioned in Section 2.2. None of the signals exceeded those of tablets taken from an extemporaneously opened bottle, in line with the absence of the subtle formation of DP1–DP4.

#### 3.2.3. Disintegration of Tablets

The disintegration time of tablets was investigated using a basket-rack assembly and conditions recommended by the JP monograph <6.09 Disintegration Test>. Tablets stored in the blister at room temperature and 40 °C demonstrated similar disintegration results compared to the control. After only 3 min, i.e., much before the limit prescribed in the JP monograph (30 min), all the tablets were fully broken apart, collapsed, and yielded to a suspension of drug and excipients. No solid residue remained on the basket rack. Still, based on the low aqueous solubility of DOR [11], it was important to perform a dissolution test to complement the disintegration test.

#### 3.2.4. Dissolution Test

**Figure 9:**
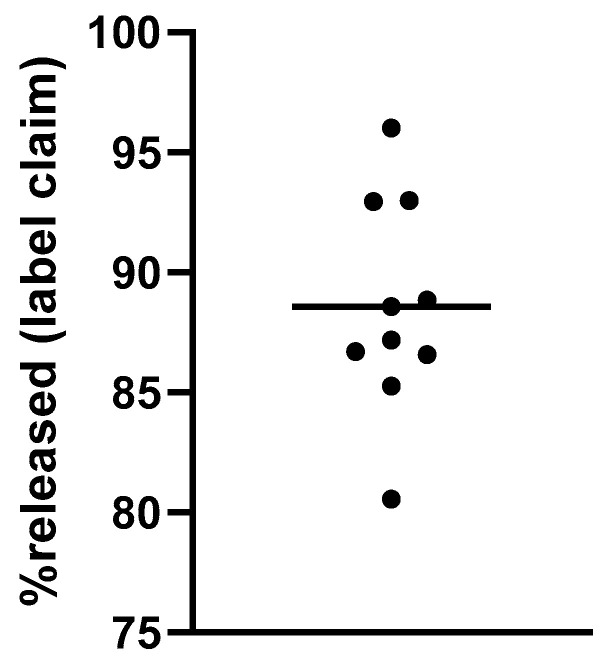
Relative amount of DOR released after 45 min of dissolution test.

Dissolution tests were carried out to confirm that the release of DOR from the repackaged tablets was appropriate. The DOR tablets consisted of an immediate release form, and for this study, the endpoint was the amounts of DOR released from tablets after 45 min. The results expressed as a percentage of the label claim are shown in Figure 9.

## 4. Discussion

### 4.1. Implications of Intrinsic Stability Studies

Intrinsic stability studies showed that, in a solution, DOR is easily amenable to degradation upon light exposure and under basic conditions. The propensity of the drug substance to degrade indicates that a change in the pharmaceutical form (by crushing, for instance) and/or of the packaging may have some implications on the drug stability.

The intrinsic stability study also enabled to propose structures of the DPs formed and to assess their mutagenic potential by the use of two different approaches (Table 1), in line with the ICH M7 recommendations [12]. The two DPs (DP2 and DP4) formed under photolytic conditions triggered in silico alerts using both approaches, in favor of a potential effect. The rule-based approach prompted an alert for the mutagenicity of DP2 and DP4 because of the presence of α,β-unsaturated carbonyl groups [16]. Among the DPs formed under basic conditions, only one, DP3, triggered an alert in the QSAR approach (Table 1).

Based on these in silico results, and in the absence of experimental data with respect to the mutagenicity of the DPs, if repackaging is to be considered, the appearance of the degradation products should be monitored with great care. Further, practitioners should be very cautious and perform an in-depth assessment if they intend to propose a liquid formulation comprising DOR.

### 4.2. Implications of Stability Testing of Tablets Repackaged in Single-Dose Unit Containers

Stability tests on the film-coated tablets showed no substantial changes in the assay and degradation profile of the drug product when repackaged in single-dose unit containers and stored at 25 °C for 6 months. The absence of evident degradation may be explained by the addition of a coating on the tablet core by the manufacturer and by the protective environment provided by the container used in the present study.

As far as the potential photodegradation of the drug in the marketed tablets is concerned, it seems that this risk is at least partially reduced by the formulation proposed by the actual manufacturer. Indeed, the European Public Assessment Report (EPAR) of Pifeltro^®^ states that “three batches were exposed to light as defined in the ICH Guideline on Photostability Testing of New Drug Substances and Products. The results indicated there were no significant changes in chemical or physical characteristics when compared with the control samples” [7]. Still, if repackaging of the tablets is contemplated, the authors recommend using UV protective blisters and a thorough follow-up of the formation of DPs under light exposure (see Section 4.1).

Furthermore, as the drug is amenable to base-catalyzed hydrolysis, the moisture content should be kept as low as possible. Thus, the following recommendations are stated by the manufacturer of Pilfetro^®^: “Store in the original bottle and keep the bottle tightly closed in order to protect from moisture. Do not remove the desiccant.” [7]. Thus, if repackaging in single dose units is needed, the authors recommend using USP class A single-unit containers as described in the USP monograph <671> CONTAINERS—PERFORMANCE TESTING [17] or equivalent and a thorough follow-up of the DPs (see Section 4.1).

In any case, the results and recommendations provided in this article may not transpose to other conditions, and the practitioner’s willingness to repackage Pifeltro^®^ tablets must balance the risk of potential change in key performance attributes of the tablets with the need to limit dispensing errors by proposing single dose units.

## 5. Conclusions

In this study, the physicochemical stability of DOR under stress conditions and changes in critical quality attributes of repackaged tablets comprising DOR stored under formal stability conditions were investigated. Under stress conditions, DOR was found particularly amenable to degradation by photolysis and basic hydrolysis. The structures of the four main degradation products formed under these conditions were analyzed using liquid chromatography coupled with high-resolution mass spectrometry.

The knowledge of DOR’s degradation product structures may be helpful in the context of other studies, for instance, in the context of the design of new pharmaceutical forms comprising DOR. Furthermore, the formal stability study of the tablets showed that repackaging in appropriate single-dose unit containers may not substantially impact some critical quality attributes (drug content and dissolution) but that an assessment of the formation of DPs should be the primary focus.

## Figures and Tables

**Table 1:** Name of compounds, conditions of formation, structures, and results of in silico testing.

Name	Condition of Formation	Structure of the Compound	In Silico Alert (Rule-Based Approach)	In Silico Alert (QSAR Approach)
DOR	NA	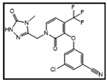	Negative	Positive
DP1	Basic hydrolysis	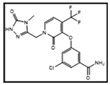	Negative	Negative
DP2	Photolysis	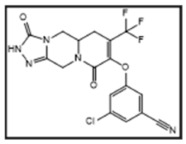	Positive	Positive
DP3	Basic hydrolysis	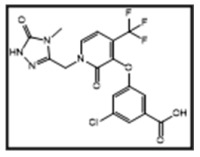	Negative	Positive
DP4	Photolysis	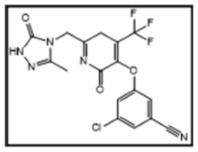	Positive	Positive

## Data Availability

The original contributions presented in the study are included in the article/Supplementary Materials, further inquiries can be directed to the corresponding authors.

## Supplementary Materials

The following supporting information can be downloaded at: https://www.mdpi.com/article/10.3390/pharmaceutics16080977/s1, Table S1: Stress conditions applied to DOR, Table S2: Validation parameters for DOR quantification, Table S3: Protocols for DOR stability studies, Figure S1: Fragmentation pattern of DP3, Figure S2: LC-HRMS^2^ of DOR, Figure S3: Zoomed LC-HRMS^2^ of DOR, Figure S4: Zoomed LC-HRMS^2^ of DOR, Figure S5: LC-HRMS spectra of DP1, Figure S6: LC-HRMS^2^ spectra of DP1, Figure S7: LC-HRMS3 of DP1 (444->427->), Figure S8: LC-HRMS spectra of DP3, Figure S9: LC-HRMS^2^ spectra of DP3, Figure S10: LC-HRMS^2^ spectra of DP3 (445->427->), Figure S11: LC-HRMS spectra of DP2, Figure S12: LC-HRMS^2^ spectra of DP2, Figure S13: LC-HRMS^2^ spectra of DP2, Figure S14: LC-HRMS spectra of DP4, Figure S15: LC-HRMS^2^ spectra of DP4.
