# Supplementary material for: Physicochemical Stability of Doravirine (Pifeltro®): Characterization of Main Degradation Products and Assessment of Stability of Tablets Repackaged in Single-Dose Unit Containers
Source: Pharmaceutics. 2024 Jul 24;16(8):977. doi: 10.3390/pharmaceutics16080977 (PMC11359146; doi:10.3390/pharmaceutics16080977)
Supplement: Supplementary file 1 [file pharmaceutics-16-00977-s001.zip › pharmaceutics-3095295-supplementary.pdf]

Figure S1: Fragmentation pattern of DP3

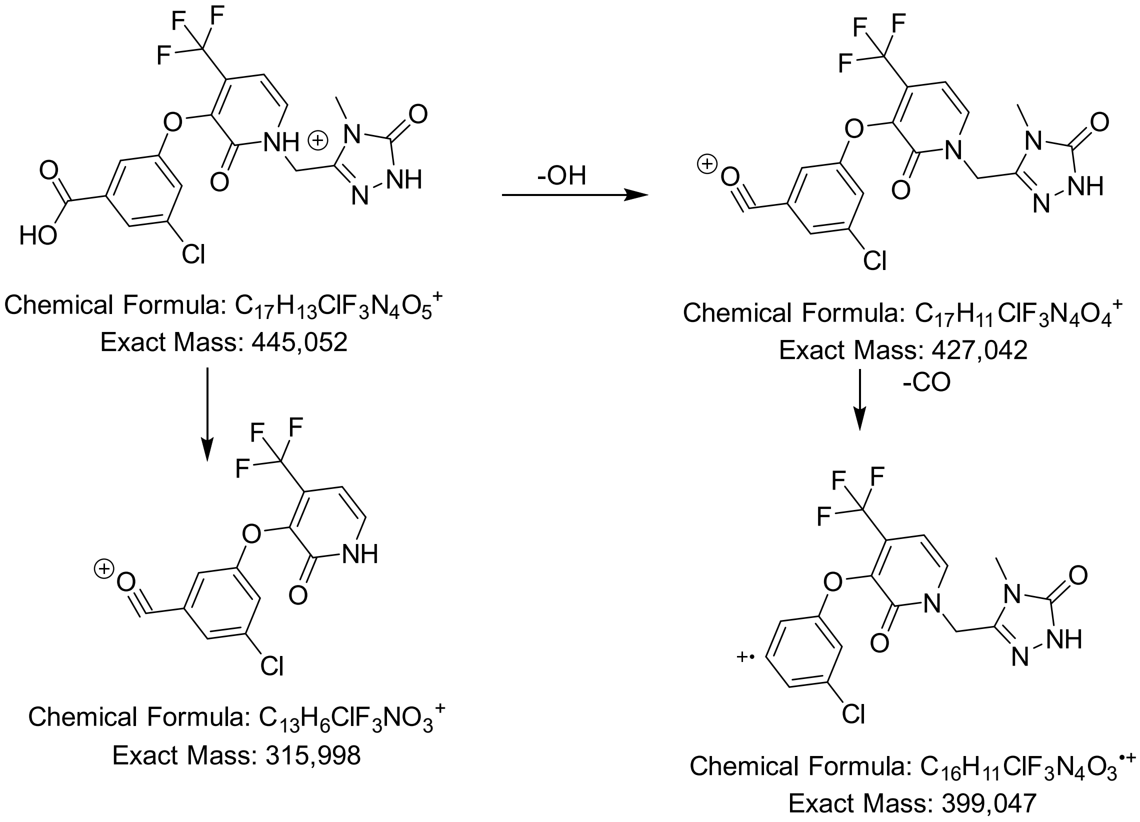

Figure S2 : LC-HRMS² of DOR

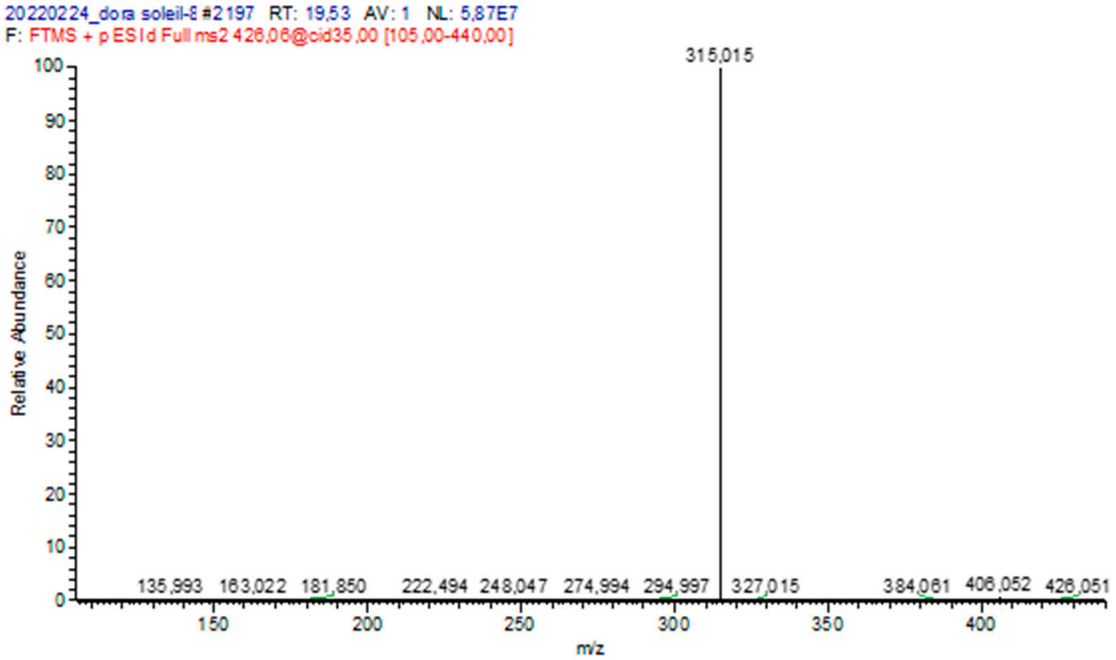

Figure S3 : Zoomed LC-HRMS^2 of DOR

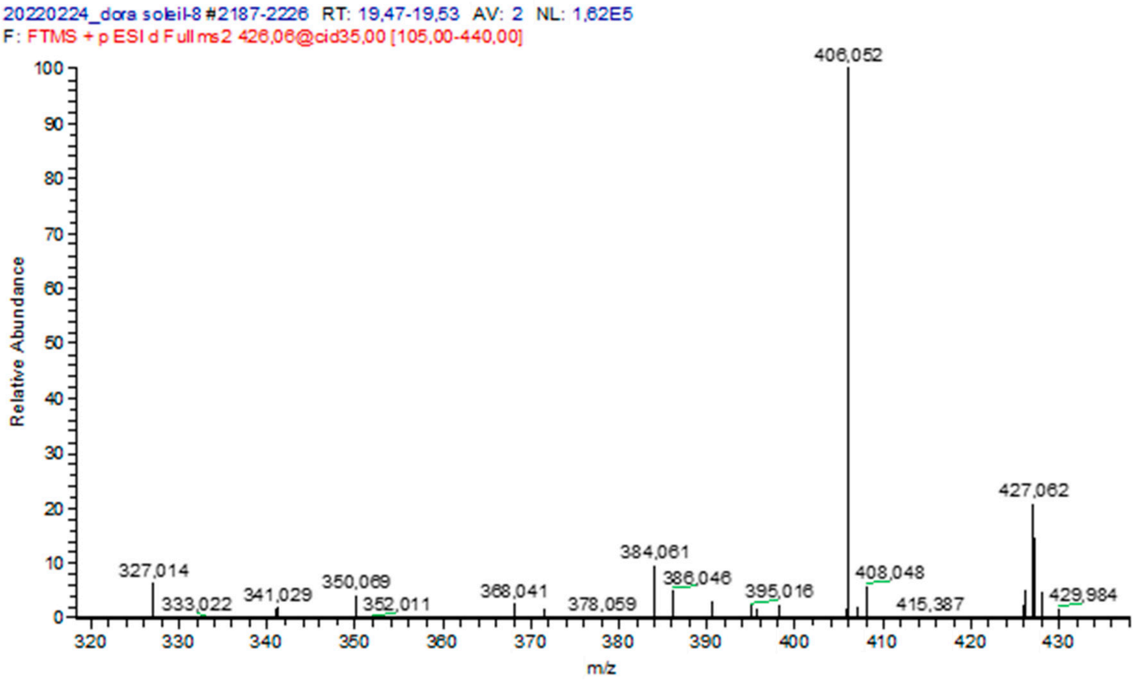

Figure S4: Zoomed LC-HRMS^2 of DOR

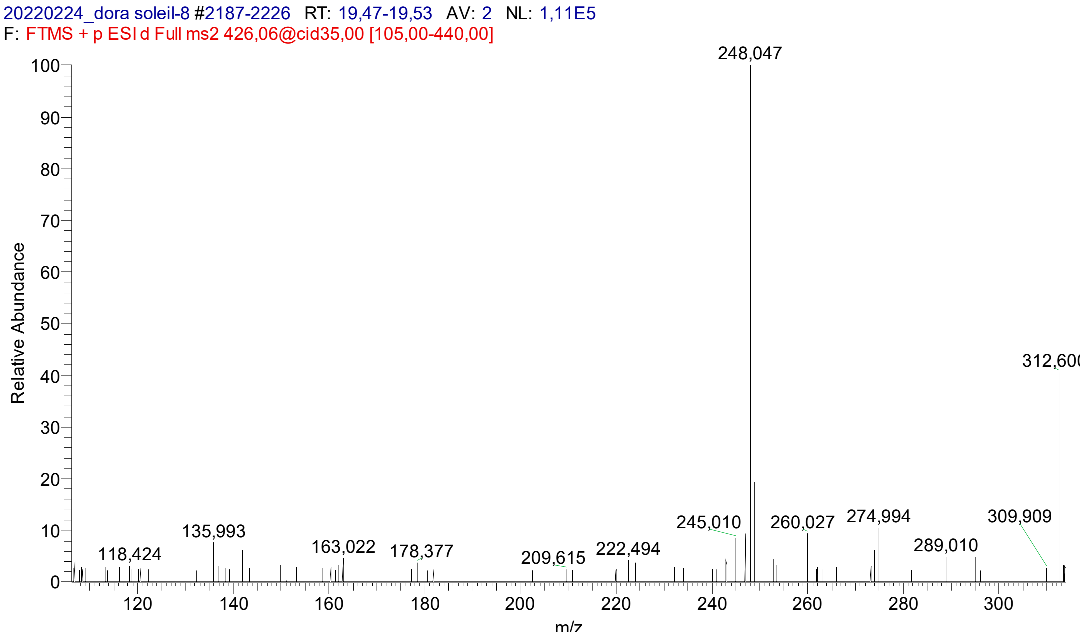

Figure S5: LC-HRMS spectra of DP1

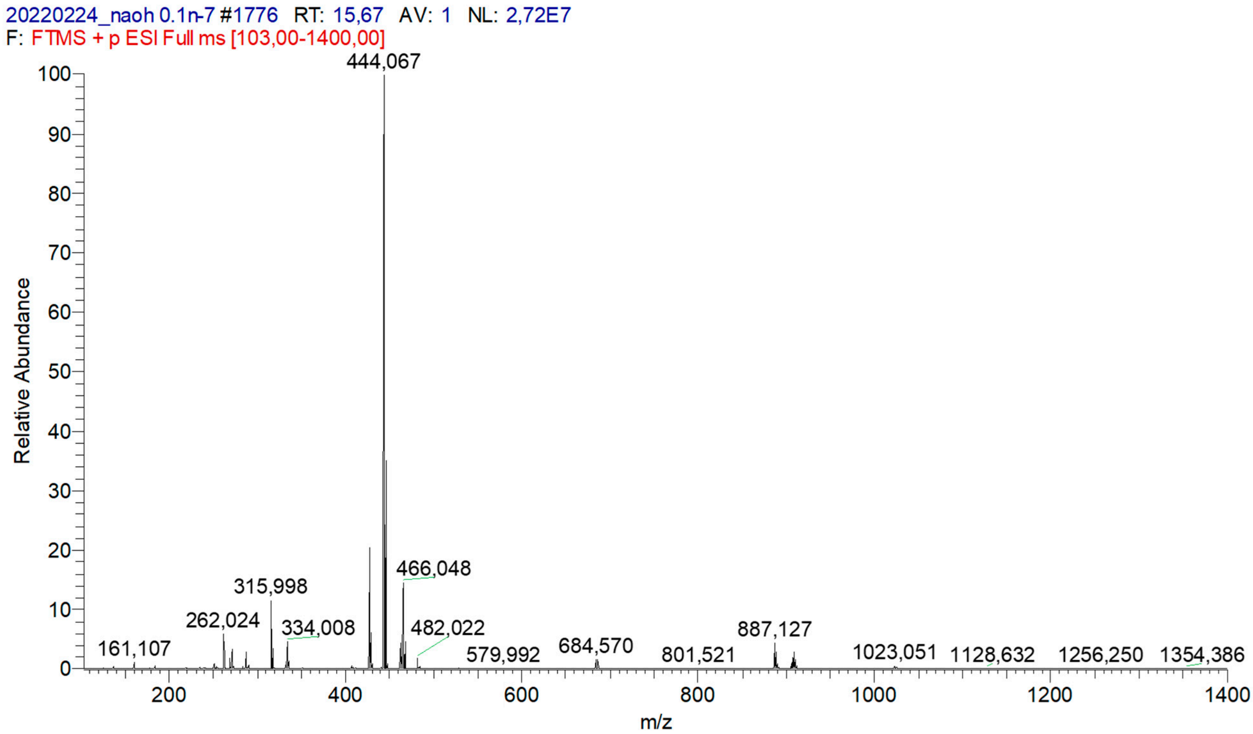

Figure S6 : LC-HRMS^2 spectra of DP1

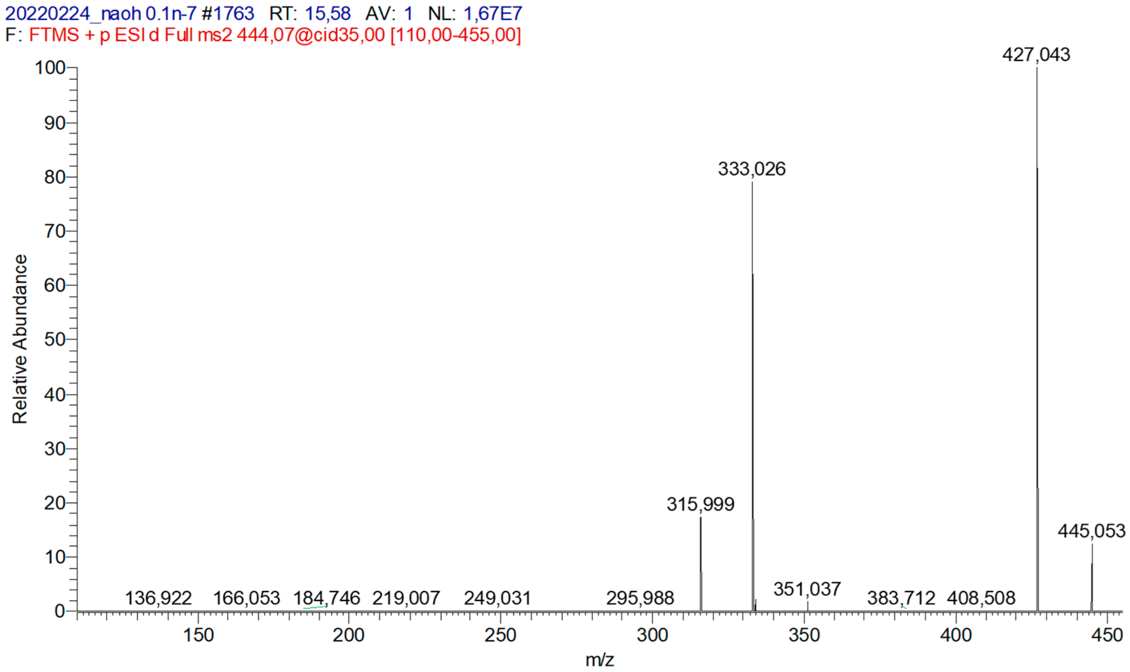

Figure S7: LC-HRMS^3 of DP1 (444->427->)

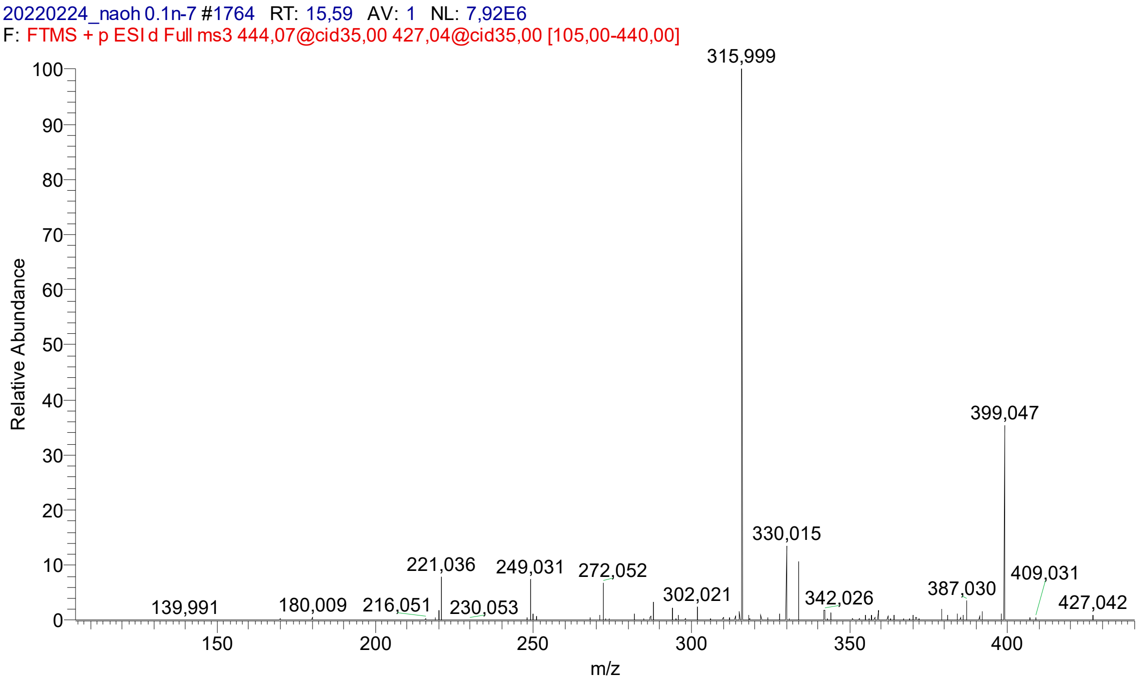

Figure S8: LC-HRMS spectra of DP3

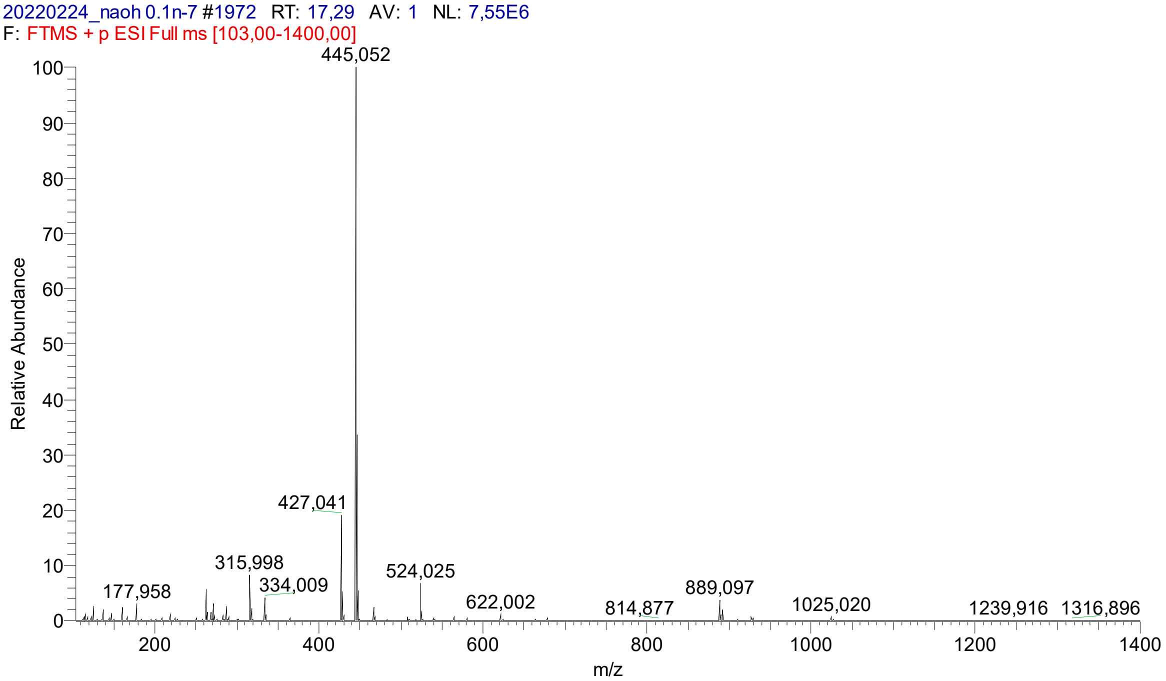

Figure S9: LC-HRMS² spectra of DP3

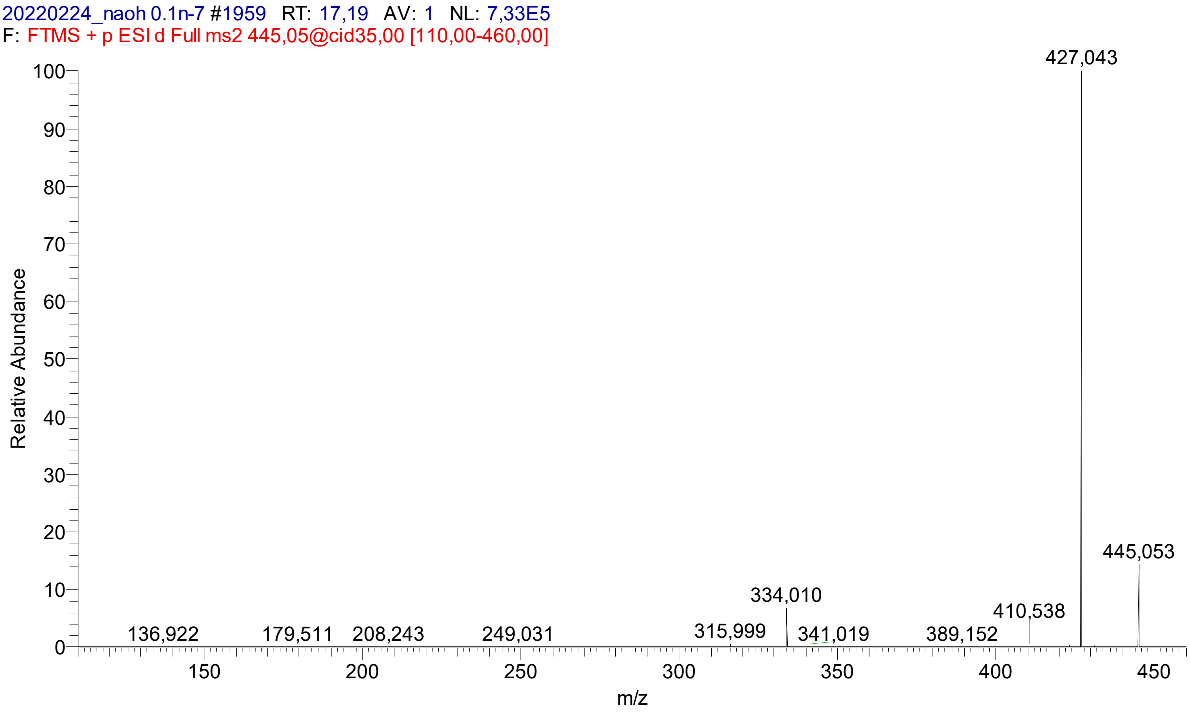

Figure S10: LC-HRMS² spectra of DP3 (445->427->)

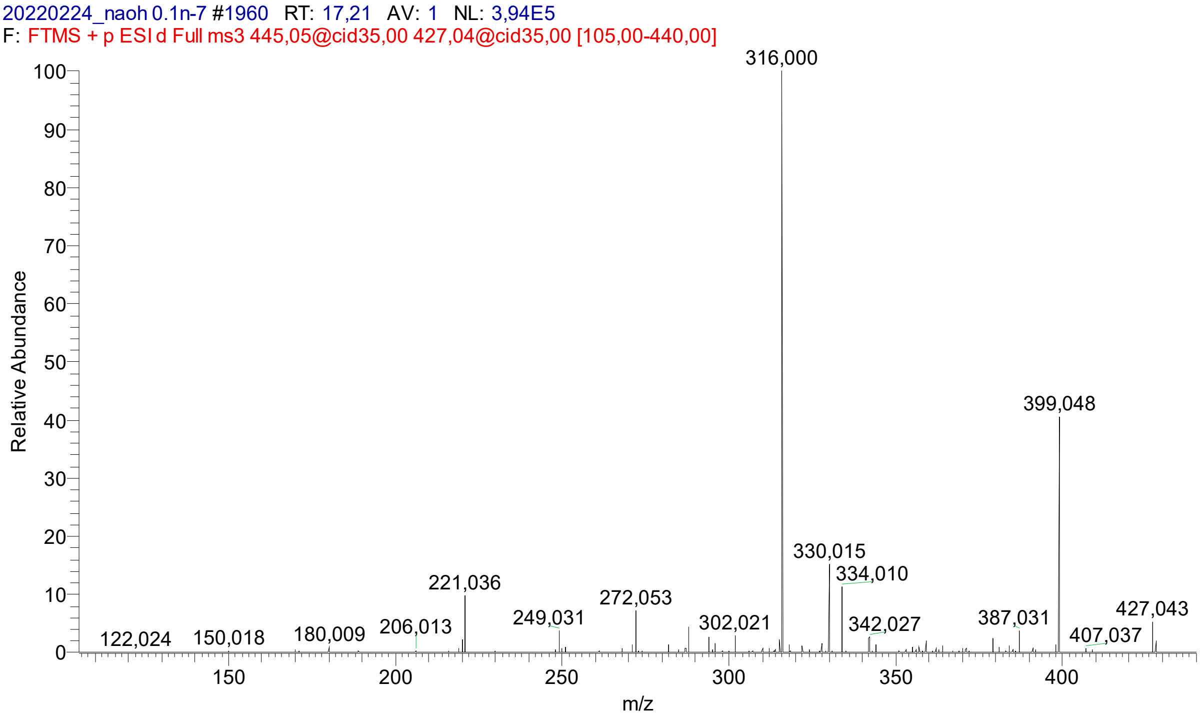

Figure S11: LC-HRMS spectra of DP2

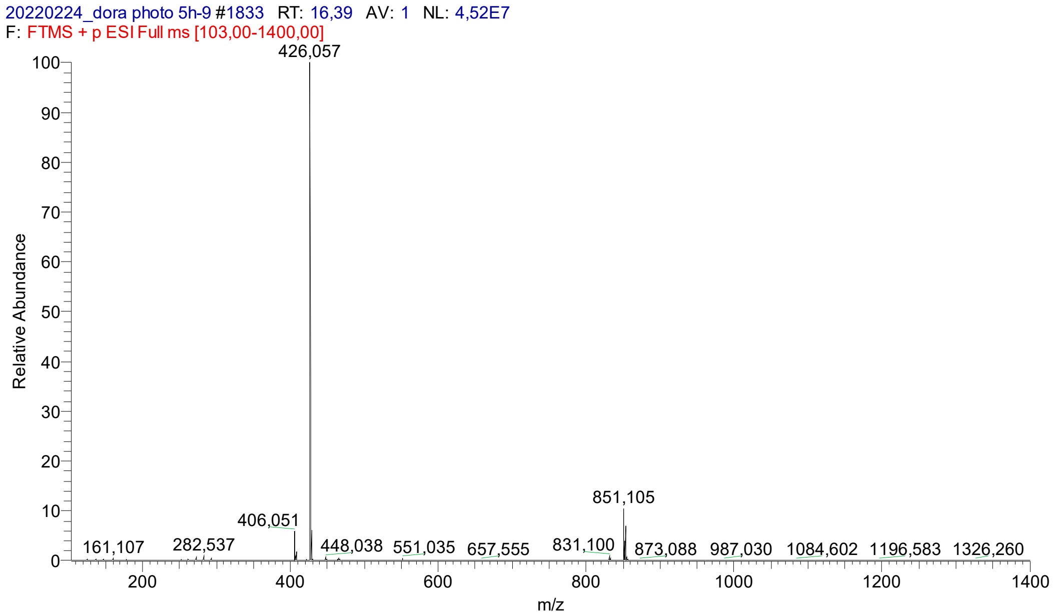

Figure S12: LC-HRMS² spectra of DP2

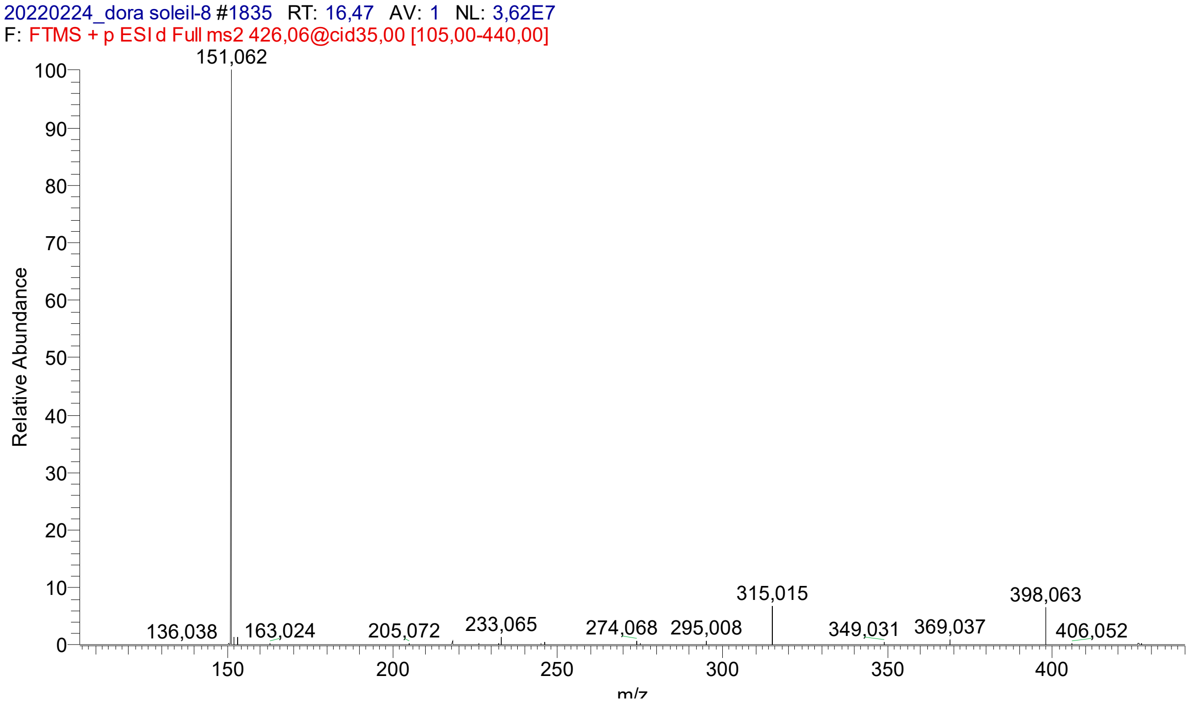

Figure S13: LC-HRMS^2 spectra of DP2

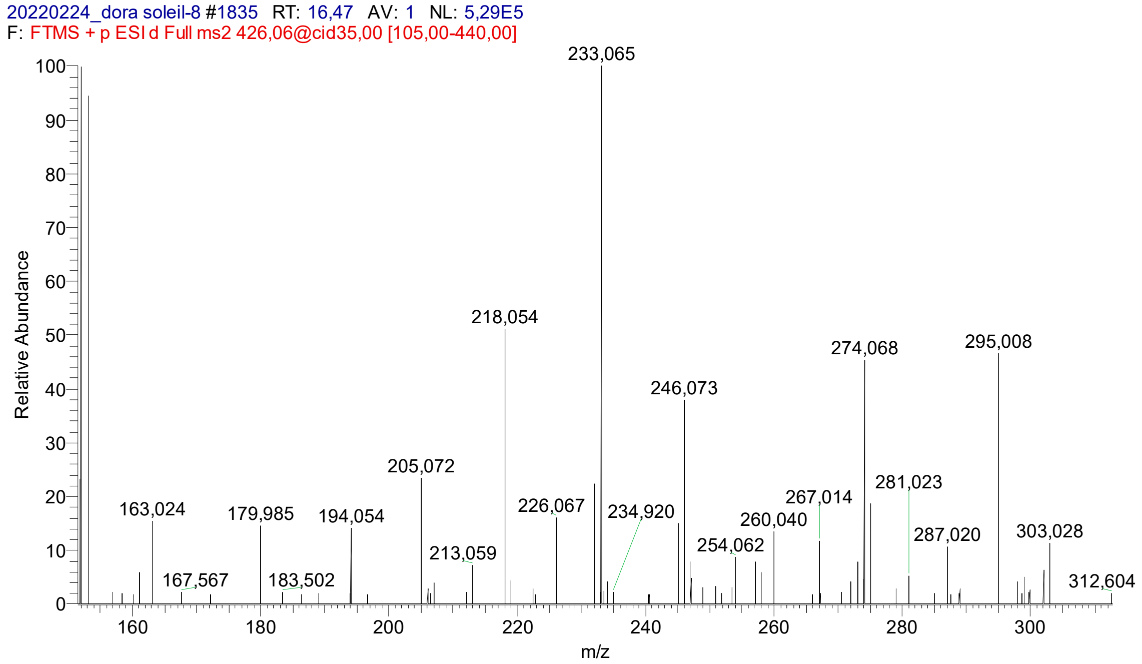

Figure S14: LC-HRMS spectra of DP4

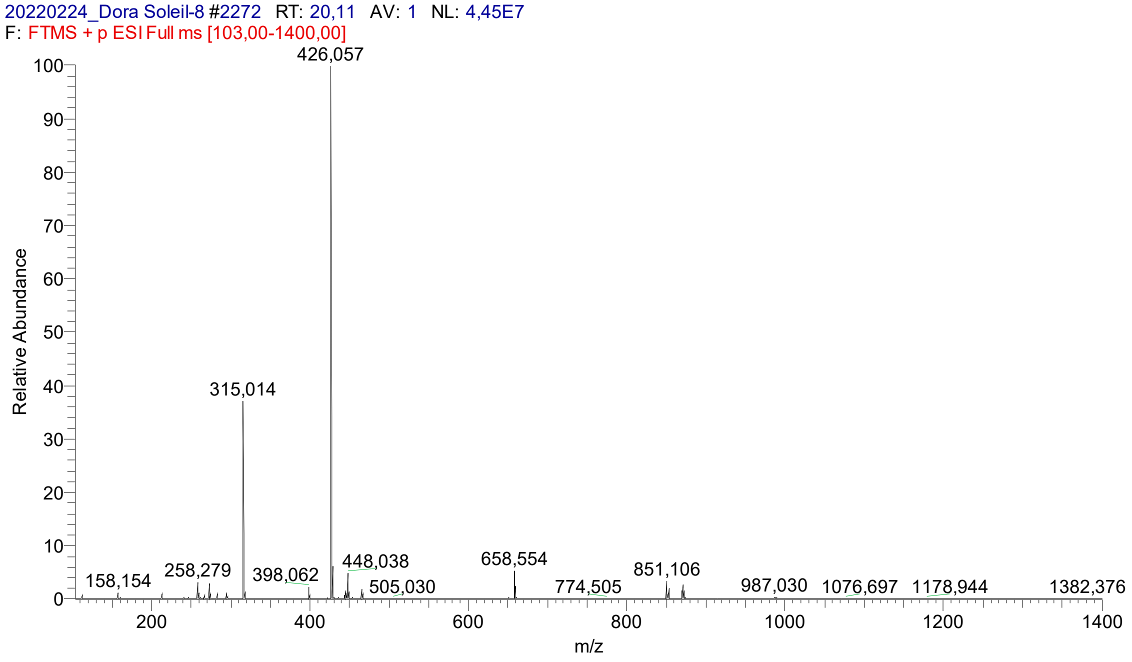

Figure S15: LC-HRMS² spectra of DP4

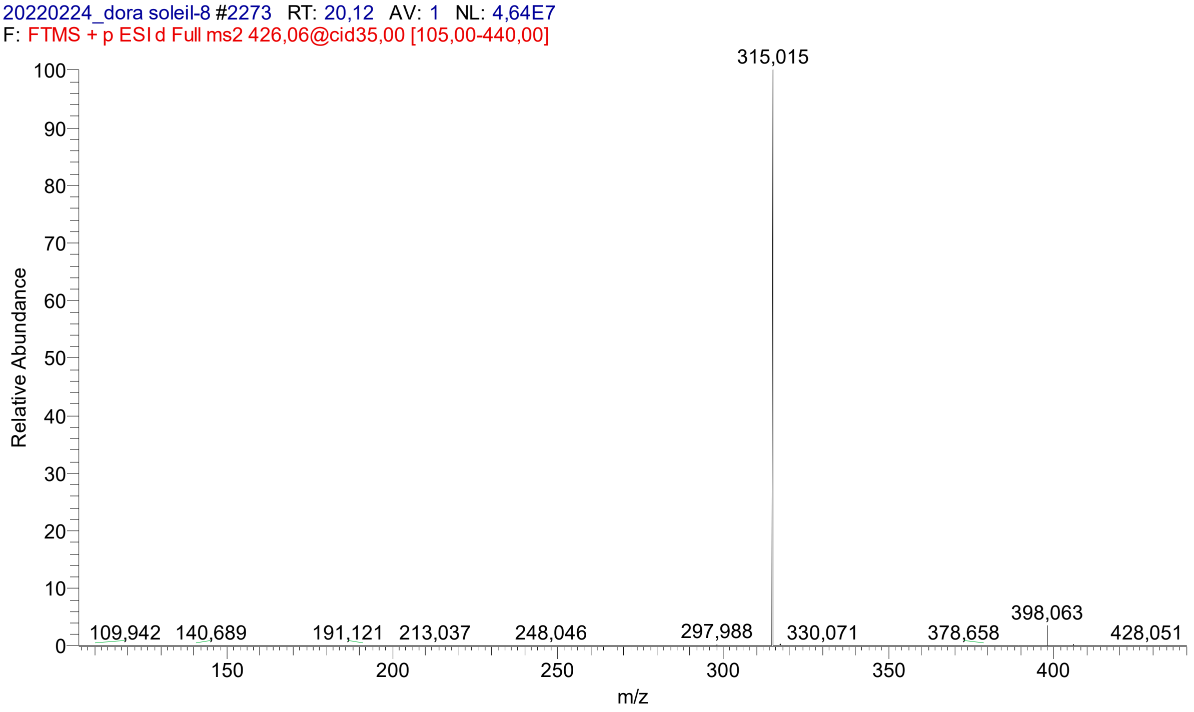

Table S1: Stress conditions applied for DOR

Stress conditions	Diluting solvent	Temperature (°C)	Duration (Hour)
Acid	0.1 N	RT	H0,H12, H24, H48
Basic	0.1 N	RT	H0,H12, H24, H48
Photolysis (1.50 W.m-2 at 420 nm)	H_2O	RT	H0, H0.5,H1,H1.5,H2
Oxidation	3.5% H_2O_2	RT	H0,H12, H24, H48
Thermal	H_2O	80	H0,H12, H24, H48

H: hour; RT: room temperature

Table S2: Validation parameters for DOR quantification

Parameters	*Results*
Linearity	
Drug: Concentration range (µg mL^{-1})	[17.5 à 32.5]
Slope	1.231
SD of Slope	0.016
Intercept	- 0.244
SD of Intercept	0.780
Correlation coefficient (r)	0.988
LOD (µg mL^{-1})	0.025
LOQ (µg mL^{-1})	0.080
Precision (RSD%, n=6)	
Inter-day	1.34
Intra-day	1.23
Accuracy	
Drug: % Recovery	99.9
SD	1.30
Stability of solution (24h) (n=6)	-1.4%

Table S3: Protocols for DOR stability studies

	Storage condition	Timeline for content variation analysis (Day)	Total impurities
Repackaged DOR tablets in PVC blister	20°C ± 2°C	D0, D1, D4, D7, D21, D30, D60, D90, D180	D0, D1, D4, D7, D21, D30, D60, D90, D180
DOR tablets In the bottle opened once daily	20°C ± 2°C	D0, D1, D4, D7, D21, D30, D60, D90, D180	D0, D1, D4, D7, D21, D30, D60, D90, D180
DOR tablets out of the bottle	20°C ± 2°C	D0, D1, D4, D7, D21, D30, D60, D90, D180	D0, D1, D4, D7, D21, D30, D60, D90, D180
DOR tablets out of the bottle	25°C, 60% Residual humidity	D0, D1, D4, D7, D21, D30, D60, D90, D180	D0, D1, D4, D7, D21, D30, D60, D90, D180
DOR tablets out of the bottle	40°C	D0, D1, D4, D7, D21, D30, D60, D90, D180	D0, D1, D4, D7, D21, D30, D60, D90, D180
